# Wakeful resting and memory retention: a study with healthy older and younger adults

**DOI:** 10.1007/s10339-018-0891-4

**Published:** 2018-10-30

**Authors:** Markus Martini, Laura Zamarian, Pierre Sachse, Caroline Martini, Margarete Delazer

**Affiliations:** 1 University of Innsbruck, Innrain 52, 6020 Innsbruck, Austria; 2 Medical University of Innsbruck, Anichstraße 35, 6020 Innsbruck, Austria

**Keywords:** Wakeful resting, Age differences, Interference, Memory retention, Memory consolidation

## Abstract

Studies indicate that a brief period of wakeful rest after learning supports memory retention, whereas distraction weakens it. It is open for investigation whether advanced age has a significant effect on the impact of post-learning wakeful rest on memory retention for verbal information when compared to a cognitively demanding distraction task. In this study, we examined (1) whether post-learning rest promotes verbal memory retention in younger and older adults and (2) whether the magnitude of the rest benefit changes with increasing age. Younger adults and older adults learned and immediately recalled two consecutive word lists. After one word list, participants rested wakefully for 8 min; after the other list, they solved matrices. Memory performance was again tested in a surprise free recall test at the end of the experimental session. We found that, overall, younger adults outperformed older adults. Also, memory retention was higher following a wakeful rest phase compared to distraction. A detailed analysis revealed that this wakeful rest benefit was significant for the older adults group, whereas the younger adults group retained a similar amount of information in both post-encoding conditions. We assume that older adults can profit more from a wakeful rest phase after learning and are more prone to distraction than younger adults. With increasing age, a short break immediately after information uptake may help better retain the previously learned information, while distraction after learning tends to weaken memory retention.

## Introduction

The period between learning and recall critically affects memory performance (Müller and Pilzecker 1900). Evidence exists that a brief period of rest after learning leads to lower forgetting rates than working on a task (Alber et al. 2014; Cowan et al. 2004; Craig et al. 2015; Dewar et al. 2007; Mercer 2015). Recent findings indicated that post-encoding distraction has a detrimental effect on subsequent memory performance regardless of whether distractors are similar or dissimilar to the learning content (Dewar et al. 2012a, b). In other words, forgetting can be induced by any mentally effortful post-encoding distraction task, irrespective of its content (Dewar et al. 2007). This view is supported by studies in different populations (amnesics: Alber et al. 2014; healthy older adults: Dewar et al. 2012a, b; Alzheimer’s disease patients: Dewar et al. 2012a, b; healthy younger adults: Mercer 2015; children: Martini et al. 2018), with different learning materials (visuo-spatial: Craig et al. 2015; verbal: Dewar et al. 2012a, b) and post-encoding distraction tasks (games: Brokaw et al. 2016; perceptual spot-the-difference: Dewar et al. 2012a, b; vocabulary learning: Mercer 2015). Moreover, various post-encoding interventions have shown a negative effect on memory retention (transcranial magnetic stimulation, or blocking of protein synthesis; see McGaugh 2015).

It has been suggested that memories take time to get consolidated, i.e. transferred into long-term memories, becoming less prone to distraction (Robertson 2012). It is assumed that memories are susceptible to interference immediately after acquisition (Wixted 2004). Consequently, reducing interference and providing a wakeful rest period should support memory consolidation and retention. However, recent studies with healthy younger adults indicated that post-encoding resting does not necessarily lead to higher delayed memory performances (Varma et al. 2017) and that memory retention is affected by the post-encoding phase only under certain conditions. It has been, for example, shown that rich autobiographical retrieval/future imagination after learning has a detrimental effect on the consolidation of recently acquired episodic memories (Craig et al. 2014). It has also been shown that wakeful resting after learning has an effect on direct forgetting (Schlichting and Bäuml 2017).

Most central for the current study, knowledge about the impact of a brief period of post-encoding rest in contrast to distraction across different age groups is scarce. Our brain changes across the lifespan and these changes often coincide with age-related alterations in cognitive task performance (Dennis and Cabeza 2008). For instance, with increasing age, grey matter and white matter losses in prefrontal cortex, parietal lobes, and specific parts of the medial temporal lobes are found to be related to a decrease in episodic memory, reasoning, working memory, and processing speed (e.g. Rodrigue and Raz 2004; Persson et al. 2006; Stebbins et al. 2002; Grady et al. 1998). Additionally, studies directly comparing older adults to younger adults point to complex patterns of hyper- and hypoactivation in different task-related brain regions (e.g. occipital cortex and prefrontal cortex, Grady et al. 1994), alterations in neural network switching (e.g. between a task-related brain state and a resting-related brain state; Pinal et al. 2015), and changes in neurotransmitter release (e.g. striatal dopamine; e.g. Bäckman et al. 2000) in older age.

Against this background, it is of relevance to test age-dependent differences in the effect of post-encoding rest on memory retention. Regarding this, first evidence was found by Craig et al. (2016) who investigated younger and older adults with a virtual route learning task. They showed that, while pointing accuracy was lower in older adults than in younger adults, both age groups significantly profited from a 10-min wakeful rest period compared to distraction (for fMRI data with an object–location association memory task see Kukolja et al. 2016).

The outline above indicates that (1) results in healthy younger adults diverge due to task manipulations and that (2) knowledge about cross-age differences in the magnitude of post-encoding rest benefits, compared to distraction, is scarce (Craig et al. 2016; Kukolja et al. 2016). Accordingly, the present study aimed at investigating (1) whether an 8-min post-encoding rest period, compared to a distraction condition, promotes verbal memory retention in younger and older adults and (2) whether the magnitude of post-encoding rest benefit, compared to distraction, changes with increasing age. In our study, healthy younger and older adults had to retain and immediately recall two word lists. We used a crossover design with participants being randomly assigned to two presentation orders. The first group (order 1) was required to wakefully rest for 8 min after having learned a first word list (rest condition), and to solve matrices for 8 min after having learned a second word list (distraction condition). The second group (order 2) received the exact opposite condition order, i.e. first distraction and then rest. Both groups were presented with a surprise delayed free recall test at the end of the experimental session. We hypothesised to find (1) lower retention rates in older adults, and (2) that resting, compared to distraction, would lead to higher retention rates in both age groups. We also expected that active rehearsal after encoding would lead to a better recall in all conditions and in both age groups.

## Method

### Participants

Fifty older participants and forty younger participants were tested. Both younger and older participants were recruited from the same socio-economic background from acquaintances or through advertisement. Some older participants were recruited in cooperation with a local association that offers a variety of different activities (e.g. physical and cognitive training) and events for adults of 60 years of age and over. Inclusion criteria were no prior neurological, medical, or psychiatric conditions which may affect cognition as indicated by an informal interview and an education level of at least obligatory school (min. 8 years). Prior to the experimental session, older participants responded to the Mini-Mental State Examination (MMSE) to screen for cognitive impairment (cut-off = 27). Eighteen older participants were excluded from analyses as they performed under the MMSE cut-off score of 27. The final sample consisted of thirty-two older participants (25 females, age: *M* = 69.41 years, SD = 5.94, age range = 57–80 years; MMSE: *M* = 28.41, SD = 1.10). Participants in the younger group consisted of forty university students (32 females, age: *M* = 21.02 years, SD = 2.28, age range = 18–29 years). Groups were comparable in terms of gender distribution, *χ*^*2*^ (*df* = 1, *N* = 72) = .00, *p* = 1.^1^

### Materials and procedure

**Fig. 1:**
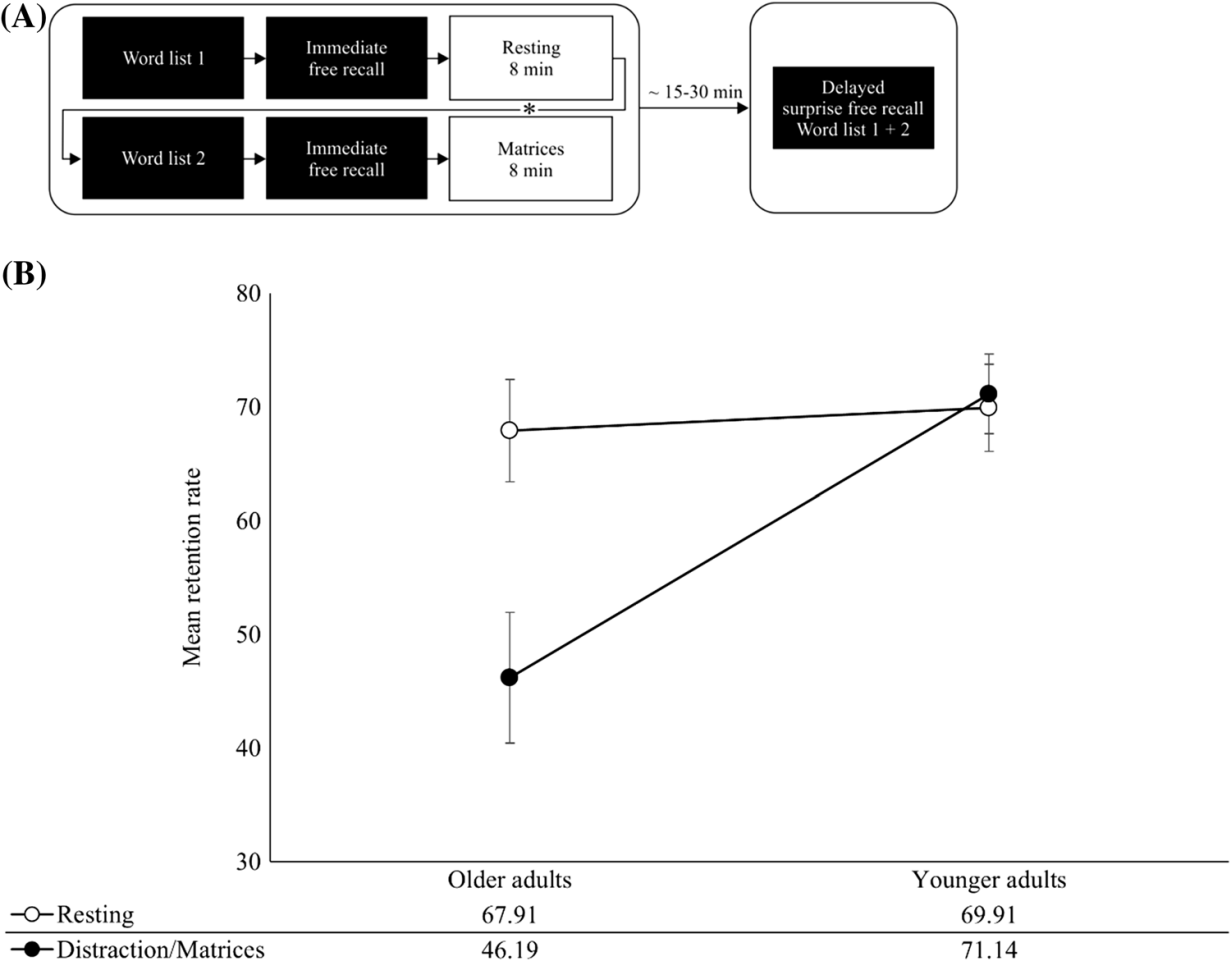
**A** Schematic illustration of the experimental design (order 1: first rest condition and then distraction condition). Conditions were counterbalanced (*). ~ 15 to 30 represents the temporal interval from immediate recall to delayed recall (first list: ca. 30 min; second list: ca. 15 min). For details see text. **B** Retention rates in rest and distraction conditions for older and younger adults. Error bars depict standard errors of the mean

Figure 1A illustrates the basic experimental procedure (Brokaw et al. 2016; Dewar et al. 2012a, b; Varma et al. 2017). Participants were required to (1) retain a first word list; (2) immediately recall the words of this list; (3) perform an 8-min post-encoding condition, where they either rested wakefully or completed a distraction task; (4) retain a second word list; (5) immediately recall words of this second list; (6) perform either a distraction task or a rest condition; and (7) finally complete a surprise free recall test. In sum, all participants performed two learning tasks, one followed by rest, the other followed by distraction. Order of word lists and post-encoding conditions (rest and distraction) was counterbalanced within both age groups.

Each word list consisted of 15 semantically unrelated German nouns and was taken from the verbal learning and memory test (Helmstaedter et al. 2001). Words were presented once, sequentially in the middle of the computer screen (Times New Roman, 100, black characters against a white background). Duration of stimulus presentation and interstimulus interval (blank white screen) were age specifically varied (older: 1000 ms/word, 1500 ms interstimulus interval; younger: 500 ms/word, 750 ms interstimulus interval). Word list presentation started after the question “Ready?” which was also displayed on the screen. An image of a writing hand presented in the middle of the computer screen indicated that participants should recall words in any order they wanted. Participants noted words on a white sheet of paper (one for each word list). Recall time was limited (older: 90 s; younger: 60 s).^2^ After the immediate recall, participants either rested wakefully or solved matrices (distraction condition).

During the rest condition, participants were asked to relax quietly with their eyes closed in the darkened testing room. The experimenter did not leave and also rested. During the distraction condition, participants were required to solve matrices (older: standard progressive matrices, Raven 1958; younger: advanced progressive matrices, Set II, Raven et al. 1998). The matrices measure abstract reasoning. Participants are presented with several items of geometric patterns. Each item consists of a target pattern with a missing part in the bottom right corner. Participants have to select the missing part out of several alternatives. All participants were instructed to solve as many items as possible. The main reason for applying the matrices was that mental resources are continuously bound by the progressive character of the task, in addition to the ease to understand explanations. At the same time, matrices are visuo-spatial in nature, thus minimising interference with the previously learned word lists. Following each post-encoding condition, participants were asked to answer two questions: (1) “How often did you think about the words?” and (2) “How often did you consciously rehearse the previously learned words?”. Participants could answer by selecting one of 7 alternatives (from 1 = “not at all” to 7 = “very often”). All participants went through a probe phase prior to the main experiment where stimulus presentation and recall were trained with five words (which were semantically unrelated to the word lists of the main experiment). At the end of the experimental session, a surprise free recall test took place. Participants were asked to write down as many words from both lists as possible in any order they wanted. Recall time was limited (older: 180 s; younger: 120 s). The recall phase was followed by the question “Did you expect a surprise recall test at the end of the experimental session?”.

## Results

We compared age groups with regard to retention rates in the rest and distraction conditions (Fig. 1B). We calculated for each word list a retention rate by dividing the number of words recalled during delayed recall by the number of words recalled during the immediate recall, separately for the rest and distraction conditions.

We conducted a mixed ANOVA on these retention rates with post-encoding condition (rest and distraction) as within-subject factor, and order (order 1: first rest condition and then distraction condition; order 2: first distraction condition and then rest condition) and age group (older and younger) as between-subjects factors. Results indicated a significant main effect of age group, *F*(1, 68) = 7.95, *p* < .006, $\eta^{2}_{p}$ = .11, with older adults (*M* = 57.05%, SD = 21.90) obtaining overall lower retention rates than younger adults (*M* = 70.52%, SD = 18.76). The main effect of condition was also significant, *F*(1, 68) = 6.05, *p* < .016, $\eta^{2}_{p}$ = .08, as were the interaction between condition and order, *F*(1, 68) = 4.61, *p* < .035, and the interaction between condition and age group, *F*(1, 68) = 7.74, *p* = .007. Other results were not significant, *p’s* > .1. Overall, retention rates for items followed by the rest condition (*M* = 69.02%, SD = 24.70) were higher than retention rates for items followed by the distraction condition (*M* = 60.05%, SD = 29.77). An investigation of the significant condition*order interaction by means of post hoc contrasts indicated that this difference (retention rates: rest condition > distraction condition) was significant for participants performing order 2 (i.e. first distraction condition and then rest condition), *F*(1,37) = 8.33, *p* = .006, $\eta^{2}_{p}$ = .18, but not for participants performing order 1, *p* > .1 (i.e. first rest condition and then distraction condition; see Table 1). We also carried out an investigation of the significant condition*age group interaction by means of post hoc contrasts. Results indicated that the difference between conditions (retention rates: rest condition > distraction condition) was significant for the older participants, *F*(1,31) = 10.05, *p* = .003, $\eta^{2}_{p}$ = .25, while younger participants performed comparably accurately in both conditions, *p’s* > .1 (see Fig. 1B).

**Table 1:** Condition- and order-specific retention rates (%)

	Post-encoding condition	
	Rest	Distraction
	*M* (SD)	*M* (SD)
Order 1 (rest–distraction)	64.20 (27.22)	63.79 (26.18)
Order 2 (distraction–rest)	73.34 (21.67)	56.71 (32.63)

A further mixed ANOVA with post-encoding condition (rest and distraction) as within-subject factor, and order (order 1 and order 2) and age group (older and younger) as between-subjects factors was performed separately on scores given to question 1 (“How often did you think about the learned words?”) and scores given to question 2 (“How often did you consciously rehearse the learned words?”). Results of the analysis carried out for question 1 indicated a significant main effect of age group, *F*(1, 68) = 14.39, *p* < .001, $\eta^{2}_{p}$ = .17, and a significant main effect of condition, *F*(1, 68) = 58.29, *p* < .001, $\eta^{2}_{p}$ = .46. Other results were not significant, *p’s* > .1. Similar results were found in the analysis performed for question 2. The main effect of age group, *F*(1, 68) = 42.49, *p* < .001, $\eta^{2}_{p}$ = .20, and the main effect of condition, *F*(1, 68) = 37.29, *p* < .001, $\eta^{2}_{p}$ = .35, were significant. Other results were not significant, *p’s* > .1. In sum, older adults reported having thought and rehearsed the words more often than younger adults. Overall, people reported having thought and rehearsed the words more often following the rest condition than following the distraction condition (see Table 2).

**Table 2:** Age-specific and post-encoding condition-specific answers to the questions whether participants thought about (question 1) or consciously rehearsed (question 2) the presented words

	Question 1 (thought)	Question 2 (rehearsed)
	*M* (SD)	*M* (SD)
Older adults^a^	2.86 (1.29)	2.63 (1.40)
Younger adults^a^	1.86 (0.91)	1.52 (0.76)
Rest condition	3.18 (1.91)	2.64 (1.85)
Distraction condition	1.43 (1.05)	1.39 (1.04)

^a^The group mean is computed by collapsing the two post-encoding conditions together

Finally, we performed a Spearman rank-order correlation analysis for the two age groups separately between retention rates and scores obtained in questions 1 and 2. This analysis was carried out for the rest and distraction conditions separately. Results were not significant, *p’s* > .1, indicating no relation between memory performance and retention strategies. Correlations were also not significant when groups were collapsed.

Fifteen older adults and eleven younger adults indicated that they had expected a surprise free recall test, *χ*^*2*^ (*df* = 1, *N* = 72) = 2.89, *p* = .089. Independent *t*-tests showed no significant differences in retention rates between participants expecting a surprise free recall test and those who did not, *p’s* ≥ .10.

## Discussion

The present study aimed at investigating age-related differences in the impact of a brief period of rest after learning, in contrast to distraction (here, solving matrices), on the recall of verbal memory material. Overall, older adults retained fewer words than younger adults, indicating a memory decline with increasing age (Li 2002; Sander et al. 2012). Importantly, post-encoding rest and distraction differently affected memory retention in the two age groups. While older adults and younger adults performed comparably in the rest condition, distraction after learning significantly affected memory retention of the older participants. These novel findings support previous studies with healthy older adults. For instance, Dewar et al. (2012a, b) found that verbal memory retention over 7 days was better when the immediate recall was followed by 10 min of wakeful rest than when it was followed by a spot-the-difference task. Our results partially support the findings of Craig et al. (2016), who investigated the impact of a brief period of wakeful rest across younger and older adults with a visuo-spatial learning task. They found that pointing accuracy in a virtual spatial navigation task was affected by a post-encoding perceptual spot-the-difference game when compared to a wakeful rest condition in both older and younger adults. In our study, we found a detrimental effect of distraction in the older group, but not in the younger group. Findings of studies with healthy younger adults are inconsistent—while some studies reported a supportive effect of post-encoding rest (Brokaw et al. 2016; Mercer 2015), others reported no beneficial effect of rest on memory retention (Varma et al. 2017; Martini et al. 2017). The latter studies indicate that wakeful rest might not be a necessary prerequisite for episodic memory consolidation. For instance, in a study with healthy young adults, Varma et al. (2017) found that memory retention was not affected by a distractor (n-back) task even when the complexity of the task was increased. Varma et al. (2017) assumed that post-encoding cognitive engagement probably has no interfering effect when the task has minimal demands on semantic processing and episodic memory supported by the hippocampus. Accordingly, memory consolidation can take place in parallel to task processing. In our study, participants solved matrices which have been related to hippocampus functioning (Colom et al. 2013; Zhu et al. 2017). Based on the assumption that hippocampal structures are relevant to post-encoding memory consolidation (Dewar et al. 2007) and that answering matrices involves the hippocampus, we should have found a detrimental effect of the distraction condition on memory retention in both age groups. However, this was only found in older adults, while younger adults were unaffected by the distraction task. One possible explanation for our results is that younger participants built memory representations of higher strength/quality than older adults, and were consequently less prone to distraction (de Zubicaray et al. 2011; McGaugh 2015; Paller and Wagner 2002; Robertson 2012; Wixted 2004). Indeed, the ability to retain newly acquired information seems to decrease with increasing age, accompanied by hypoactivation in memory-relevant brain regions, compensatory hyperactivation of brain areas relevant to attention and executive control, and reductions in interregional connectivity (see, for example, Lindenberger 2014; Park and Reuter-Lorenz 2009). As our results indicate, this decrease in retaining newly acquired information after distraction is particularly pronounced in older participants. Though deficient/slow consolidation of memory contents is a reasonable explanation for age-related memory declines after distraction, alternative hypotheses have to be taken into account. As repeatedly shown in different domains (Hinault et al. 2017; Lemaire 2016), younger and older individuals differ from each other in the development, choice, and application of cognitive strategies. Older adults are less efficient and less flexible in strategic choice than younger adults due to an age-related decline of frontal lobe functioning (Daselaar and Cabeza 2013). Memory contents supported by efficient memory strategies (e.g. visual imagination, stories) may be more resistant to interference in the distraction condition, while strategies may be less relevant in the rest condition. Since younger individuals are more efficient in developing and applying strategies, they should experience a smaller decline in long-term retention after distraction than older adults. In the present study, we did not investigate memory strategies. Future research may elucidate the effect of individual strategies in memory acquisition and memory retention on the benefit associated with a rest condition.

Finally, we found that resting supported memory retention in older adults only for the second word list. These results indicate that 8-min resting after encoding word list 1 (rest condition) was not sufficient to shield memory representations against interference through the following learning unit (distraction condition). Thus, in older adults, position and length of the resting phase seem to be of relevance. Our findings cannot be directly compared with other studies using a within-subject design (e.g. Dewar et al. 2012a, b) as, even though post-encoding conditions were counterbalanced, order effects were not explicitly reported.

We should acknowledge some limitations. First, we tested almost four times more female participants than male participants. We cannot, therefore, make any conclusion about possible gender differences in memory retention after wakeful rest and distraction. Second, our study leaves open whether the education level might have been a modulating factor. Previous studies have found that higher education is positively related to cognitive functioning throughout adulthood and is negatively associated with the risk of dementia (e.g. Anstey and Christensen 2000; Hall et al. 2007). However, there has been also evidence that participants with lower education engaging frequently in cognitive activities show significant compensatory benefits for episodic memory (Lachman et al. 2010). Finally, we did not administer other cognitive tasks. Therefore, the association between wakeful rest benefit and other cognitive abilities such as interference inhibition, working memory, or divided attention remains to be investigated. Future studies might also consider the effects of encoding strategies, the complexity of the learning material, the length of the learning phase, and the specific time point of resting on memory retention across the lifespan.

To conclude, we found that older adults profited from a brief period of wakeful rest and were more affected by post-encoding distraction than younger adults. Our results suggest that a brief period of wakeful rest can support memory retention and that this strategy is especially effective in older age. This could be taken into account when planning cognitive interventions and counselling for older adults with a special focus on memory.

## Footnotes

1 We performed a mixed ANOVA with post-encoding condition, order, and gender as factors to consider possible gender effects on retention rates. Age was not entered into this analysis as the two age groups had a comparable gender distribution. We found a significant condition*gender interaction, but no other significant results. Post hoc contrasts indicated that female participants profited significantly from the rest condition relative to the distraction condition, whereas male participants did not show any difference between conditions. These results should be considered with caution as the sample size of the two gender groups differed relevantly (57 females vs. 15 males).

2 In a pilot study, we tested immediate memory performance of 5 younger adults under different stimulus durations and interstimulus intervals. According to the results of this pilot study and the results by Ecker et al. (2015), we decided to use a stimulus presentation duration of 500 ms and an interstimulus interval of 750 ms. Based on the existing findings that psychomotor speed is reduced in elderly people (e.g. Salthouse 1991, 1994), we decided to double the stimulus and interstimulus presentation durations for the older group. Moreover, older participants received 30 s more time than younger participants to recall words for a better comparability of memory performance between the two age groups.
